# Study on age-dependent pre-existing 2009 pandemic influenza virus T and B cell responses from Chinese population

**DOI:** 10.1186/s12879-017-2215-1

**Published:** 2017-02-10

**Authors:** Jin Lv, Zhen-Yong Ren, Ying-Ying Zhang, Yun-E Liu, Jun Gao, Kun Yao, Dan Feng, Zhen-Yuan Li, Xin Feng, Yun-Xi Liu, Na Jia

**Affiliations:** 1 The General Hospital of PLA Rocket Force, 16 Xin Jie Kou Wai Street, Hai-Dian District, Beijing, 100088 People’s Republic of China; 2 State Key Laboratory of Pathogen and Biosecurity, Beijing Institute of Microbiology and Epidemiology, 20 Dong-Da Street, Fengtai District, Beijing, 100071 People’s Republic of China; 3 Chinese PLA General Hospital, 28 Fu-Xing Road, Hai-Dian District, Beijing, 10853 People’s Republic of China; 4 Beijing Center for Disease Prevention and Control, Beijing, 100013 People’s Republic of China; 5 National Development and Reform Commission Hospital, Beijing, People’s Republic of China

**Keywords:** Pandemic influenza, Memory B cell, IFN-γ^+^ T cell, ELISpot, Age-dependent

## Abstract

**Background:**

The outbreak of the 2009 H1N1 influenza pandemic (H1N1pdm) affected thousands of people in Mexico and the United States, and spread rapidly throughout the world from April 2009 to July 2010. To explore the age-specific prevalence of seroprotection against H1N1pdm infection, we estimated pre-existing humoral and cellular immunities of residents in Northern China against H1N1pdm and seasonal H1N1 virus in an age-dependent manner.

**Methods:**

Anonymous serum samples were collected from 1425 to 1434 adult healthy individuals before and after the pandemic outbreak, and then grouped by birth year 1913–1990. The antibody titers of H1N1pdm and seasonal H1N1 were determined using microneutralization (MN) assays, and the proportion of seropositive was estimated based on the year of birth. Separately, another 63 blood samples were collected in 2006 and prepared for analysis of virus specific memory B and IFN-γ^+^ T cells using the ELISpot assays.

**Results:**

The prevalence of pre-existing H1N1pdm-specific sero-antibodies in the elderly population (>60 years old) was 7.8%. The younger group, aged 19 to 60 years, exhibited a significant increase in seropositivity for H1N1pdm after the pandemic (4.9% before pandemic and 18.9% after pandemic, *p* < 0.05). The prevalence of H1N1pdm specific MBCs before the pandemic in the elderly (>60 years) and younger populations (<60 years) was 38% (8/21) and 48% (20/42), respectively (*p* = 0.6). The IFN-γ^+^ T cell responses to the pandemic and seasonal viruses were significantly lower in the elder group than those in the younger group (<60 years) (*p* < 0.05).

**Conclusions:**

Pre-existing serum antibodies and memory B cells against H1N1pdm were low in all age group, whereas diminished memory T cell responses to this virus were observed in the elderly population both before and after the pandemic.

**Electronic supplementary material:**

The online version of this article (doi:10.1186/s12879-017-2215-1) contains supplementary material, which is available to authorized users.

## Background

In April 2009, an outbreak of H1N1 influenza initially hit Mexico and rapidly spread to other countries and regions of the world. On June 11, 2009, World Health Organization (WHO) declared the first state of emergency about the influenza pandemic in 21^st^ century [1]. Historically, there were four worldwide pandemics of influenza which had caused millions of death. The first influenza pandemic in 1918 which caused by H1N1 strain killed 40 to 50 million people [2]. In 1957, the prevalent strain of influenza virus H1N1 in human abruptly disappeared and replaced by a new reassortant of influenza virus H2N2, which contained three new segments from the avian source and maintained the other five segments from the H1N1 strain of 1918 lineage [3]. In 1968, the circulating influenza virus H2N2 subtype transformed to H3N2 subtype by reassortment of the novel hemagglutinin (HA) and polymerase PB1 segments [4]. In January 1976, a novel virus subtype, A/New Jersey/76 H1N1, was identified in an outbreak of respiratory disease occurred among soldiers returning to an Army base in Fort Dix, New Jersey. However, this virus subtype did not escape from the base [5]. In 1977, separate emergence of another H1N1 virus successfully propagated [6], and then co-circulated with 1968 H3N2 subtype in human population globally. The 2009 pandemic H1N1 virus (H1N1pdm) was antigenically similar to pre-1950 influenza strains [7] and A/New Jersey/76 H1N1 strain [8].

The prevalence of pre-existing memory B cell against 2009 H1N1pdm in human population has been rarely evaluated. The ELISpot assay had been developed to count antigen-specific memory B cells in human blood by Crotty et al. [9]. These detected antigen specific memory B cells satisfied the canonical surface phenotype of human memory B cells: CD19^+^CD20^+^Ig^+^CD27^+^ [9]. This method had been widely used to assess the immunological memory of B cells response to various infectious diseases [10].

As estimated in China and other countries, overall age-standardized H1N1pdm cumulative incidence varied significantly by age with the highest in children 5–19 and 0–4 years old [11]. This age distribution was different from the seasonal influenza which mostly infected the elderly population [12]. In the present study, we evaluated neutralization antibodies against H1N1pdm as well as recent circulating seasonal H1N1 viruses in serum samples collected before and after the pandemic. The samples were further resorted by birth year to estimate the age-specific H1N1pdm infection. In addition, we examined H1N1pdm and seasonal H1N1 specific memory B and IFN-γ^+^ T cells frequencies using ELISpot assay in healthy individuals, whose blood had been collected in 2006, and aimed to evaluate the potential connections of pre-existing cellular immunities and age-dependent H1N1pdm influenza infections.

## Methods

### Ethics statement

The research involving human materials was approved by Institutional Review Board at the China Center for Disease Control and Prevention. The study was performed anonymously and with the written informed consent provided by the participants.

### Sero-epidemiological study before and after the pandemic influenza A (H1N1) 2009 virus outbreak

#### Serum sample collections

We recruited adult volunteers (≥20 years old) from 1) health examination individuals from a big health examination center in Beijing 2) employee annual health check-up from one national department in Beijing China. H1N1pdm firstly emerged in May and reached peak in November, 2009, according to influenza surveillance in Beijing [13]. Serum samples from 1425 individuals pre-pandemic were collected from June, 2008 to April, 2009, and 1434 serum samples post-pandemic were collected from January to July 2010. During serum samples collection, seasonal influenza H1N1 prevailed from October 2008 to April 2009 [14], and followed with the epidemic of seasonal H3N2 from mid-July to late-September 2009 in Beijing [15]. The information of collection date, age and gender was provided as appendix in the end (Additional file 1: Table S1).

#### Viral preparation

Seasonal influenza A/Brisbane/59/2007 and 2009 pandemic influenza A/California/07/2009 viruses were kindly provided by Dr. YL Shu, Chinese National Influenza Center, and propagated in embryonated chicken eggs. The allantoic fluid containing viruses were collected, filtered and stored in aliquots at −70 °C. Virus titers were determined by EID_50_ (50% egg infectious dose), TCID_50_ (50% tissue culture infectious dose) and PFU (plaque forming units) in Madin Darby Canine Kidney (MDCK) cells.

#### Serological assays

*Microneutralization assay (MN)* As previously reported [16], heat inactivated sera were serial 2-fold diluted, and then preincubated with an equal volume of A/California/07/2009 (H1N1) or A/Brisbane/59/2007 (H1N1) influenza virus in 96 well plates. After 1 h incubation, the virus-serum mixtures were added in the monolayer of MDCK cells, and continued with incubation at 37 °C and 5% CO_2_ for another 18–20 h. The monolayer cells were washed and fixed. The presence of viral protein was detected by ELISA with the influenza NP monoclonal antibody (kindly provided by Dr. Adam Meijer., National Institute for Public Health and the Environment, Netherlands). The neutralization endpoint titer was calculated as described in detail by Rowe T et al. [16]. The antibody titer >40 was taken as equivalent of seropositivity.

*Hemagglutination inhibition assay (HI)* All sera were treated with receptor destroying enzyme (RDE) and heat-inactivation, and absorbed with the chicken erythrocytes to remove non-specific hemagglutination. The HI assay was performed as the WHO recommended protocol [17]. The HI titer was defined as the reciprocal of the last dilution of serum samples that completely inhibited hemagglutination.

A subtotal of 648 samples were tested by both MN and HI assays. Spearman’s rank correlation analysis had demonstrated a good correlation of the both assays to seasonal and pandemic influenza viruses (*f* = 0.745 and 0.786, respectively, *p* < 0.0001). Thus, MN assay was used solely to test the remained samples.

### Pre-existing human memory B cells and IFN γ^+^-T cells to pandemic influenza A (H1N1) 2009

#### Peripheral blood mononuclear cell (PBMC) samples

The PBMCs were isolated from 63 healthy residents (aged from 20 to 90 years) of a village in Northern China, collected in early November, 2006 as a part of routine influenza surveillance program (before 2006–2007 seasonal influenza epidemic). The enrolled criterion included no influenza vaccination in the past 5 years, no autoimmune disease such as systemic lupus erythematosus (SLE) or rheumatoid arthritis (RA), and without ongoing immunosuppressive treatment. PBMCs stored in liquid nitrogen were thawed and cultured in warm RPMI 1640 complete medium supplied with 10% FBS, penicillin/streptomycin and L-glutamine (HyClone, Thermo Scientific, USA) before the ELISpot assays of memory B cells and IFN γ^+^-T cells.

#### IgG^+^ memory B cell assay (MBC)

The IgG^+^ memory B cell assay, which required the stimulation of memory B cells to antibody secreting cells (ASC), was performed as previously described by Crotty et al. [9] and successfully established in our lab (Additional file 2: Figure S1). Briefly, PBMCs were cultured for 5 to 6 days in the presence of a mix of polyclonal mitogens, including pokeweed mitogen extract (kindly provided by Prof. Shane Crotty, La Jolla Institute for Allergy and Immunology, La Jolla, CA), CpG oligonucleotide ODN-2006 (SANGON, Shanghai, China), and fixed *S.aureus*, Cowan (SAC, Sigma-Aldrich, Co. St Louis. MO). To determine HA-specific MBCs, ELISpot plates (Millipore, Billerica, MA) were pre-coated overnight with influenza HA antigen (2 μg/mL) of A/California/07/2009 or A/Brisbane/59/2007 strain (eENZYME, LLC. Montgomery Village, MD) and then blocked by incubation with 2% BSA in PBS for 1–2 h at 37 °C. The stimulated PBMCs were added into coated plates as described and incubated for 6 h at 37 °C. The captured MBCs were detected by donkey anti-human IgG Fc biotin conjugated antibody (Jackson immunoresearch laboratories, Inc.), followed by streptavidin-alkaline phosphatase (ALP) (Vector Laboratories, Inc. Burlingame, CA) and developed by alkaline phosphatase substrate kit (Vector Laboratories, Inc.). The keyhole limpet hemocyanin (KLH, 2 μg/mL) (Sigma) coated in plate was considered as the negative control antigen. The donkey anti-human Ig (Jackson immunoresearch laboratories, Inc. West Grove, PA) coated in plate was used to enumerate total IgG^+^ MBCs of PBMCs. The frequency of HA-specific MBCs was defined as the proportion of HA-specific MBCs in the total IgG^+^ MBCs. The samples were scored positive if the frequency of HA-specific MBCs was higher than that of negative control coated with KLH.

#### IFN-γ^+^ ELISpot

The 96-well ELISpot plates were pre-coated with IFN-γ mAb (Mabtech, Stockholm, Sweden) and blocked with RPMI 1640 supplied with 1% BSA overnight at 4 °C. A total of 5 × 10^5^ cells/well PBMCs suspended in RPMI 1640 medium with the supplement of 2.5% BSA, penicillin/streptomycin and L-glutamine) were infected with either influenza virus A/California/07/2009 or A/Brisbane/59/2007 (suspended in media RPMI 1640 media with 2.5% BSA) at a multiplicity of infection (MOI) of 1 or 2. Plates were incubated overnight at 37 °C. The infected PBMCs were added into pre-coated plates as described previously and incubated for 6 h at 37 °C, followed by washing and detection with biotinylated secondary monoclonal antibody (7-B6-1, Mabtech) and ALP substrate kit (Vector Laboratories). Background was determined in cultures containing media alone, or allantoic fluid from mock-infected eggs at the same gestational age. Phytohemagglutinin (PHA, 10 μg/mL) was used as the positive control. The numbers of spots in each well were counted by an automated CTLtd ELISpot plate reader (Cellular Technology Ltd. Shaker Heights, OH) and results were presented as spot-forming cells (SFC) in per million PBMCs.

### Statistic analysis

Statistical analysis was performed in SPSS statistical package version 19.0 (SPSS, Chicago, IL, USA). Basic means and percentages were calculated. Data of HA-specific MBCs and IFN-γ^+^ T cells were presented as dot plot of the raw data overlaid by box-and-whisker plot (median, first and third percentile, range). Paired *t*-test was used to compare pre-existing B or T cell immunity between seasonal and pandemic influenza infections. *P* value <0.05 was considered as statistical significance.

## Results

A total of 1425 and 1434 un-paired serum samples were collected pre- and post-pandemic, respectively. Separately, another set of PBMCs from 63 individuals were collected before the pandemic and used for the cell immunity assays (Additional file 1: Table S1).

### Birth year-dependent seroprevalence of A (H1N1)pdm 2009 and seasonal influenza

**Fig. 1:**
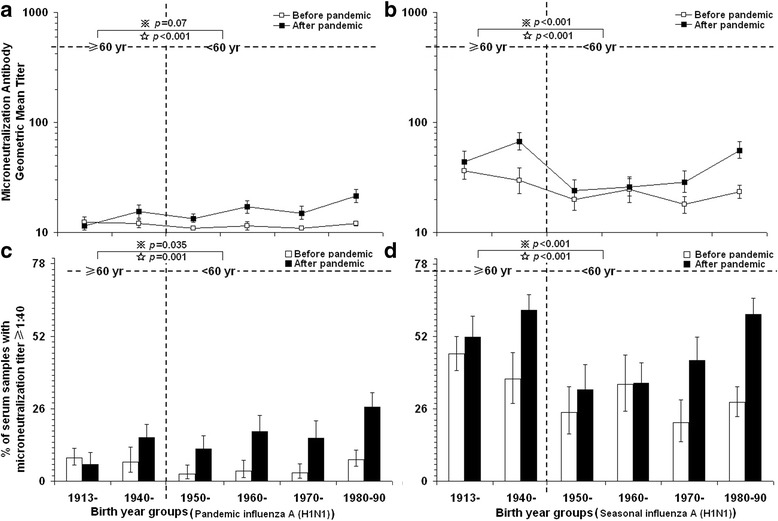
Antibody titers of A/California/07/2009 (2009 H1N1 pandemic influenza A) and A/Brisbane/59/2007 (H1N1 seasonal influenza A) viruses grouped by birth year before (from June, 2008 to April, 2009) and after the pandemic (from January to July, 2010) in Beijing, China. **a**, **b** Geometric mean titers (95% confidence intervals). **c**, **d** Percentage with microneutralization assay titers ≥40 (95% confidence intervals). Undetectable titer was set at the level of 10 for calculation of geometric mean titers. ≥60 years: equal and older than 60 years old; <60 years: younger than 60 years old. *P* value was calculated between people over and under 60 years old ※ before pandemic and ☆ after pandemic

Before the pandemic, the estimated proportion of seropositive to 2009 H1N1pdm virus was generally as low as 5.9% across the population studied (Fig. 1a and c). Although elder people over 60 years of age had a higher seroprevalence (7.8%) than younger people under 60 years (4.9%, *p* = 0.035) before the pandemic, there were no significant difference of geometric mean titers (GMT) between the elder people (12, 95% CI: 12–13) and the younger people (11, 95% CI: 11–12; *p* = 0.07). After the first wave of pandemic in Beijing, the overall seropositive against pandemic H1N1 virus increased to 16.3%. In addition, the proportion of seropositives to the 2009 H1N1pdm virus in younger people under 60 years old (18.9%) was significantly higher than the proportion in people over 60 years old (11.9%, *p* = 0.001). Moreover, the GMTs in the younger group (17, 95% CI: 16–18) were significant higher than in the elder people group (14, 95% CI: 13–15; *p* < 0.001) (Fig.  1a, c). Overall, the significant increased titers against 2009 H1N1pdm virus was observed in people born after 1950s (younger than 60 years old) (*p* < 0.05).

Differently, there were significant higher proportion of seropositives and GMTs against seasonal influenza (A/Brisbane/59/2007) in older people over 60 than in younger people under 60 years old regardless of the 2009 H1N1pdm [Positive rate: 42.1 vs. 27.3% pre-pandemic, 57.7 vs. 45.8% post-pandemic; GMT: 33 (95% CI, 29–38) vs. 22 (95% CI, 20–24) pre-pandemic, 57 (95% CI, 49–64) vs. 35 (95% CI, 31–38) post-pandemic, respectively; *p* < 0.001] (Fig. 1b, d). Furthermore, significantly increased proportions of seropositive individuals and higher microneutralization antibody titers against seasonal influenza were observed both in the elder and the younger group after the pandemic (*p* < 0.001) (Fig. 1b, d).

### Birth year-dependent pre-existing memory B cells

**Fig. 2:**
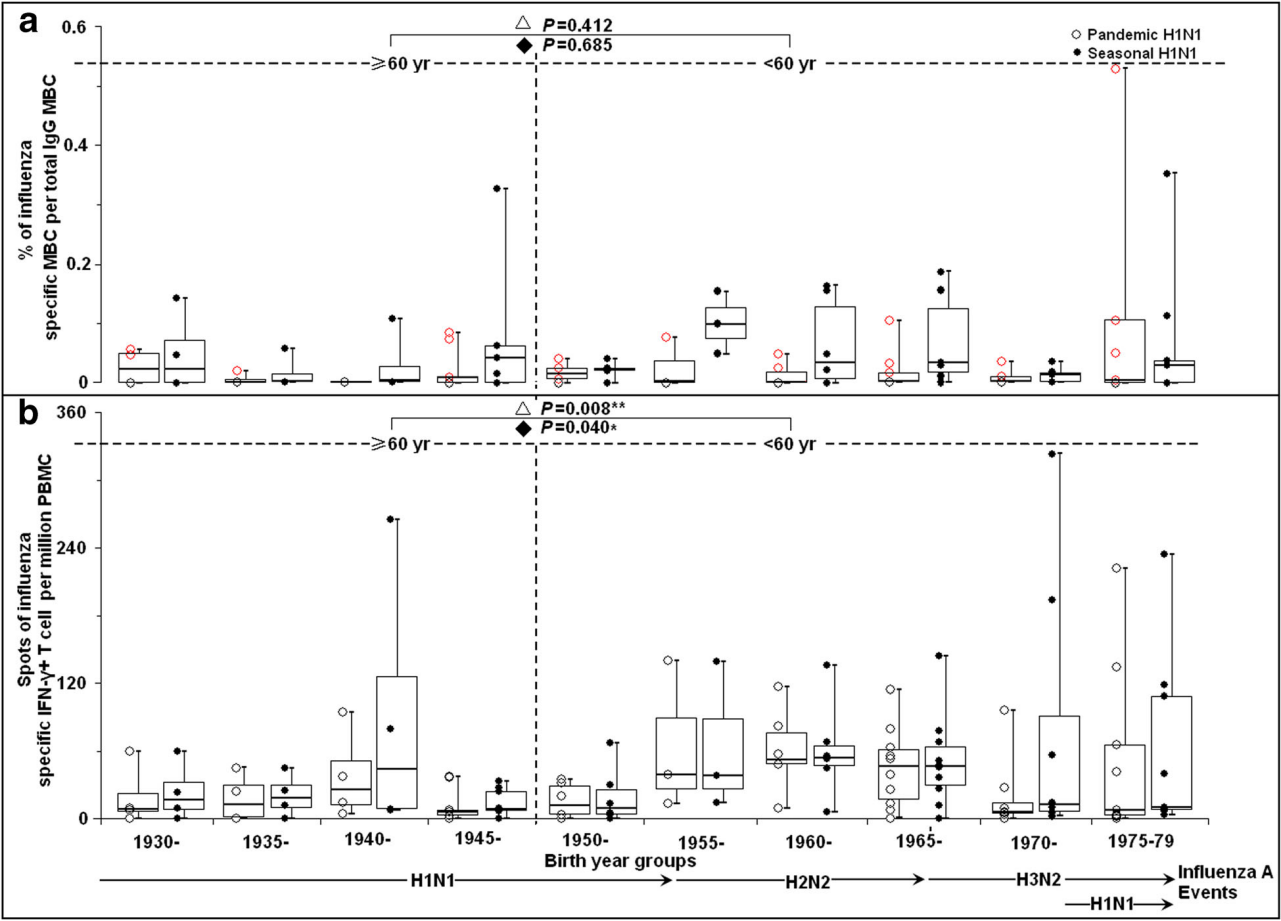
The overall response patterns of pre-pandemic influenza specific B and IFN-γ^+^ T cell immunities from the same individual against pandemic and seasonal H1N1 grouped by the year of birth. **a** Frequency of influenza hemagglutinin (HA) specific memory B cell as a percentage of the total IgG^+^ memory B cells. MBCs, memory B cells. **b** Frequency of IFN-γ^+^ T cell per million PBMCs stimulated with live influenza viruses. The data were presented as *dot plot* of the raw data, overlaid by *box-and-whisker plot* (median, first and third percentile, range). *Blank circle point* in *red* represents as the positive for HA specific MBCs of H1N1 pdm. Pandemic H1N1, A/California/07/2009 (H1N1). Seasonal H1N1, A/Brisbane/59/2007 (H1N1). The *horizontal line* in each column indicates the median value. ≥60 years: equal and older than 60 years old; <60 years: younger than 60 years old. *P* value was calculated between people over and under 60 years old △stimulate with pandemic and ◆seasonal influenza virus

The frequency profile of pre-existing MBCs from all the 63 samples (collected pre-pandemic) was exhibited as scatter plots in Fig. 2a. Screening by ELISpot assay showed that 28 samples (28/63, 44%) were positive for H1N1pdm influenza HA specific MBCs (Fig. 2a). Among the 28 MBCs positive samples (44.4%, 28/63), 8 samples (38%, 8/21) were from the individuals over 60 years old, while 20 (48%, 20/42) were from under 60 years old (Fisher Exact Test, *p* = 0.6). In general, low proportions of pandemic influenza H1N1-specific MBCs were observed across the studied groups.

For seasonal influenza, there were 33 samples (33/63, 52%) positive for HA specific MBCs, including 8 of 21 individuals (38%, 8/21) over 60 years and 25 of 42 individuals (60%, 25/42) under 60 years old (Fisher Exact Test, *p* = 0.1). Up to 0.4% of circulating IgG^+^ memory B cells were recognizing seasonal influenza (mean ± SE = 0.05 ± 0.01%) (Fig. 2a). The proportions of seasonal influenza HA specific MBCs also didn’t show any significant difference between the people over 60 and under 60 years old (*p* = 0.685) (Fig. 2a).

### Birth year-dependent pre-existing IFN-γ^+^ T cells

The frequencies of IFN-γ^+^ SFC per million PBMCs exhibited a relatively variable distribution with the stimulation of live influenza viruses (Fig. 2b). For both the viruses, the magnitudes of T cell response in the elderly participants over 60 years (seasonal influenza: mean ± SE = 28 ± 10; H1N1pdm influenza: mean ± SE = 18 ± 4) were significantly lower than those in younger participants under 60 years old (seasonal influenza: mean ± SE = 61 ± 12; H1N1pdm influenza: mean ± SE = 45 ± 8) (*t*-test, *p* = 0.008, *p* = 0.04 respectively) (Fig. 2b). Although there were no statistical differences of the proportions of IFN-γ^+^ T cell positive individuals between seasonal influenza H1N1 (91%, 57/63) and H1N1pdm (86%, 54/63) among the total subjects (Fisher Exact Test, *p* = 0.6), the overall magnitudes of IFN-γ^+^ SFC against seasonal influenza (mean ± SE = 47 ± 8) were significantly higher than those against H1N1pdm (mean ± SE = 34 ± 5) (Paired *T*-Test, *p* = 0.02).

## Discussion

Compared with other countries [2, 18–21], elderly people in China had a relatively low baseline of antibody production responding to the 2009 H1N1pdm [22, 23]. In a serosurvey of large samples (n = 2379), only 2.0% elder Chinese people had pre-existing antibodies responding to H1N1pdm virus [22]. In the present study, our data further demonstrated a relatively low frequency of pre-existing H1N1pdm virus-specific MBCs in the elderly population in China. It was not fully understood why Chinese elder people had a lower infection rate than the youth during the 2009 H1N1pdm.

The pre-existing CD8^+^ T lymphocytes (CTL) memory can mediate heterologous immunity among different subtype influenza A viruses [24]. Although recent seasonal influenza induced little cross-reactive antibody production against H1N1pdm virus [21], cellular responses might provide immune protection by targeting invariable or cross-reactive epitopes. A total of 49% of the epitopes in recent seasonal H1N1 were found totally conserved in H1N1pdm [25], furthermore, CD8^+^ T cells specific for conserved epitopes could lyse pandemic influenza infected cells in vitro [26]. One research indicated that H1N1pdm virus shared immunogenic peptides with the catastrophic 1918 H1N1 strain as well as viruses circulating prior to 1945 [27]. Cross-reactive CTL immunity between the H1N1pdm strain and the 1918 Spanish H1N1 strain might be related with the lower susceptibility to 2009 H1N1pdm in people over 65 years of age [27]. Another recent study suggested that high susceptibility of children to the pandemic H2N2 in 1957 might be more closely linked to the number of influenza exposures [28].

Our study indicated that live influenza virus (both pandemic and seasonal influenza) stimulated poor responses of IFN-γ^+^ T cells in the older population, consisting with the other studies using peptide pools stimulation [29]. We supposed the reduced T cell responses in the older group might be due to aging of the immune system and reduced responsiveness. Aging has a significant impact on CTL responses in murine model [30]. Not only naïve epitope-specific CD8^+^ T cells decline with age [31], but also TCR repertoire diversity decreases with age [32]. All these reduced T cell function in the older individuals might lead to underestimated response of magnitude in IFN-γ^+^ ELISpot assay. It seemed that early priming of CTL response prior to aging was the key for establishment of long-lasting and protective immunity.

In our IFN-γ^+^ ELISpot assay, we used the live influenza virus. It was important to understand the responds of IFN-γ^+^ T cells to live virus, because these cells were presumably reacting to processed virus from within infected antigen presenting cells. With the use of live virus, adequate epitopes through the natural infection of APCs for the activation of virus specific IFN-γ^+^ T cells could be generated [29, 33, 34]. However, it was difficult to directly assess the HA specific humoral and cellular immunity responses when using live virus to stimulate APCs. Virus activated IFN-γ^+^ T cells could produce kinds of pro-inflammatory cytokines or directly clear virus from the infected cells, thus benefit to the rapid recovery from influenza infection [35]. The influenza virus specific IFN-γ^+^ T cells responses were mainly initiated by conserved antigens (such as nucleoprotein and M protein) and therefore the ELISpot assay could detect the influenza virus with highly diverse subtypes [36].

Pre-existing sero-antibodies and memory B cells against H1N1pdm virus were low across all studied population. Although seropositives of H1N1pdm virus was higher in people over 60 years than in ones under 60 years, the difference is too small (7.8 vs 4.9%) to deduce one solid conclusion. Moreover, the GMT titers were not significantly different between two age groups. In contrast, sero-antibodies against seasonal influenza H1N1 were higher in the elder than in the younger people. The high seroantibody responses in the elderly might be the results of high infection rate in this susceptible population during the seasonal influenza season in Northern China [37]. However, seasonal HA specific MBC was not high in the older people. We proposed that an impaired memory B cell response in the elderly as observed in vaccination subjects might be one of the plausibility [38]. However the real mechanism might be much more complex. We observed that even with low numbers of seasonal H1N1-specific MBCs elderly individuals maintained higher serum antibody levels than younger people and theses were boosted after the pandemic wave. But this was not the case for pdmH1N1 where there were low padmH1N1-specific MBCs and low serum antibody levels. The pattern of repeated exposure to seasonal strains in population might be expected as one of the explanations. However, it could not fully explain the discrepant memory responses between seasonal and pandemic influenza H1N1.

There were several limitations in the present study. Firstly, the serum samples of pre and post-pandemic were not well paired, and the vaccination history and health status of participants were not identified. These might yield biased prevalence of H1N1pdm in different age groups. Secondly, the blood samples used for the serological assays and cell immunity assays were not well paired, and the number of samples used for cell immunity assay was not enough to get the statistical power. Thirdly, the lack of additional experiments to better characterized the memory T or B cell responses (such as CD45RA, CD45RO, CD4^+^IFN-γ^+^, CD8^+^ IFN-γ^+^), cytokine profile under stimulation with influenza peptides, and so on, due to the limited accessible samples, made further evaluation of immune response impossible.

## Conclusions

In conclusion, we demonstrated that the pre-existing serum antibodies and specific memory B cells against H1N1pdm were all low in studied Chinese population, whereas in the elderly population, we observed a reduced memory T cell response both against pandemic and seasonal influenza viruses pre- and post-pandemic.

## Additional files

Additional file 1: Table S1. Sample size and sex of subjects recruited in the study, China. Serum samples from 1425 individuals pre-pandemic were collected from June, 2008 to April, 2009, and 1434 serum samples post-pandemic were collected from January to July 2010. During serum samples collection, seasonal influenza H1N1 prevailed from October 2008 to April 2009, and followed with the epidemic of seasonal H3N2 from mid-July to late-September 2009 in Beijing. The information of collection date, age and gender was provided in Additional file 1. (DOC 43 kb)
Additional file 2: Figure S1. Image of total IgG^+^ and influenza hemagglutinin (HA)-specific memory B cells. Total IgG^+^ and influenza hemagglutinin (HA)-specific memory B cells were measured by a memory B-cell ELISPOT assay (seeded with 5 × 10^5^ PBMC). Cal07-HA, HA protein of A/California/07/2009 (H1N1). Bris59-HA, HA protein of A/Brisbane/59/2007 (H1N1). KLH, Keyhole limpet hemocyanin. (JPG 64 kb)

## Abbreviations

ALP: Alkaline phosphatase
APCs: Antigen presenting cells
ASC: Antibody secreting cells
BSA: Bull serum albumin
CTLtd: Cellular Technology Limited
CTL: Cytotoxic lymphocyte
EID_50_: 50% egg infectious dose
ELISA: Enzyme linked immuno sorbent assay
ELISpot: Enzyme linked immuno spot
FBS: Fetal bovine serum
GMT: Geometric mean titers
HA: Hemagglutinin
HI: Hemagglutination inhibition assay
IFN-γ: Interferon gamma
KLH: Keyhole limpet hemocyanin
MBCs: Memory B cells
MDCK: Madin Darby Canine Kidney
MN: Microneutralization
MOI: Multiplicity of infection
NP: Nucleoprotein
PB1: Polymerase proteins
PBMC: Peripheral blood mononuclear cell
Pdm: Pandemic
PFU: Plaque forming units
PHA: Phytohemagglutinin
RA: Rheumatoid arthritis
RDE: Receptor destroying enzyme
RPMI: Roswell park memorial institute
SE: Standard error
SFC: Spot-forming cells
SLE: Systemic lupus erythematosus
TCID_50_: 50% tissue culture infectious dose
WHO: World Health Organization
