# Supplementary material for: Study on age-dependent pre-existing 2009 pandemic influenza virus T and B cell responses from Chinese population
Source: BMC Infect Dis. 2017 Feb 10;17:136. doi: 10.1186/s12879-017-2215-1 (PMC5301333; doi:10.1186/s12879-017-2215-1)
Supplement: Additional file 1: Table S1. — Sample size and sex of subjects recruited in the study, China. Serum samples from 1425 individuals pre-pandemic were collected from June, 2008 to April, 2009, and 1434 serum samples post-pandemic were collected from January to July 2010. During serum samples collection, seasonal influenza H1N1 prevailed from October 2008 to April 2009, and followed with the epidemic of seasonal H3N2 from mid-July to late-September 2009 in Beijing. The information of collection date, age and gender was provided in Additional file 1. (DOC 43 kb) [file 12879_2017_2215_MOESM1_ESM.doc]

Table S1 Sample size and sex of subjects recruited in the study, China.

| **Birth year groups** | **Sero-epidemiology study** | | | | **Pre-existing B and IFN-γ^+^ T cell immunity study** | |
| --- | --- | --- | --- | --- | --- | --- |
|  | **Before the pandemic** | | **After the pandemic** | | **Before the pandemic** | |
|  | **(June,2008-April,2009)** | | **(January-July, 2010)** | | **(November, 2006)** | |
|  | **n** | **female %** | **n** | **female %** | **n** | **female %** |
| 1913- | 248 | 55 | 115 | 49 | 4 | 50 |
| 1935- | 99 | 56 | 84 | 46 | 4 | 25 |
| 1940- | 86 | 55 | 114 | 39 | 4 | 50 |
| 1945- | 64 | 46 | 191 | 41 | 9 | 44 |
| 1950- | 86 | 57 | 97 | 51 | 6 | 50 |
| 1955- | 112 | 51 | 129 | 51 | 3 | 67 |
| 1960- | 89 | 53 | 109 | 53 | 6 | 67 |
| 1965- | 79 | 48 | 114 | 47 | 10 | 40 |
| 1970- | 113 | 46 | 96 | 46 | 8 | 50 |
| 1975- | 90 | 48 | 79 | 38 | 9 | 89 |
| 1980- | 200 | 49 | 179 | 46 | - | - |
| 1985-1990 | 159 | 46 | 127 | 41 | - | - |
| **Sum** | 1425 | 51 | 1434 | 46 | 63 | 54 |
