# Supplementary figures and images for: Study on age-dependent pre-existing 2009 pandemic influenza virus T and B cell responses from Chinese population
Source: BMC Infect Dis. 2017 Feb 10;17:136. doi: 10.1186/s12879-017-2215-1 (PMC5301333; doi:10.1186/s12879-017-2215-1)

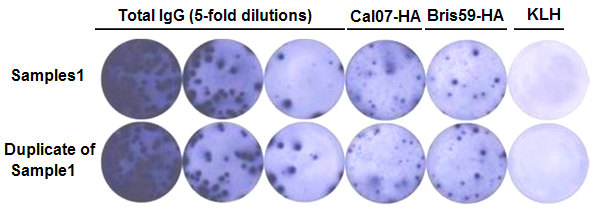

Supplement: Additional file 2: Figure S1. — Image of total IgG+ and influenza hemagglutinin (HA)-specific memory B cells. Total IgG+ and influenza hemagglutinin (HA)-specific memory B cells were measured by a memory B-cell ELISPOT assay (seeded with 5 × 10^5^ PBMC). Cal07-HA, HA protein of A/California/07/2009 (H1N1). Bris59-HA, HA protein of A/Brisbane/59/2007 (H1N1). KLH, Keyhole limpet hemocyanin. (JPG 64 kb) [file 12879_2017_2215_MOESM2_ESM.jpg]
